# Supplementary material for: Effects of High Forage/Concentrate Diet on Volatile Fatty Acid Production and the Microorganisms Involved in VFA Production in Cow Rumen
Source: Animals (Basel). 2020 Jan 30;10(2):223. doi: 10.3390/ani10020223 (PMC7070707; doi:10.3390/ani10020223)
Supplement: Supplementary file 1 [file animals-10-00223-s001.pdf]

Effects of High Forage/Concentrate Diet on Volatile Fatty Acid Production and the Microorganisms Involved in VFA Production in Cow Rumen

Lijun Wang[1,2], Guangning Zhang[2], Yang Li[2] and Yonggen Zhang[2*]

[1]College of Animal Science and Technology, Qingdao agricultural university, No. 700 of Changcheng Road, Qingdao, Shandong Province 266000, China; wlj880626@163

[2]College of Animal Science and Technology, Northeast Agricultural University, No. 600 of Changjiang Road, Harbin, Heilongjiang Province 150030, China; zgn1234@126.com ; liyang1405053@sina.com;

[*] Correspondence: zhangyonggen409@126.com; Tel) +86-451-55190840, Fax) +86-451-55190840.

Table S1 Ingredient compositions and nutrition levels of diets (% of DM)

**Ingredients, % of DM**	**Dietary frage-to-concentrate ration (F:C) (% of DM)**	
	**70:30 (F:C)**	**35:65(F:C)**
Corn silage	25.39	12.82
Alfalfa hay	0	11.19
Chinese wildrye	47.91	11.09
DDGS[1]	7.30	0
Corn bran	8.08	6.64
Cottonseed meal	0	5.82
Soybean meal	4.74	13.49
Corn starch	0	11.09
Steam flaked corn	0	15.23
Soybean hull	0	6.07
Cottonseed	0	6.04
Concentrates[2]	6.23	0
Premix[3]	0.26	0.55
Total	100	100
**Nutrient contens of diets**		
CP[1], % of DM	13.92	18.19
NDF[1], % of DM	52.43	37.10
ADF[1], % of DM	27.51	21.44
NFC[1], % of DM	28.89	37.73
Starch, % of DM	8.94	22.26
Ca, % of DM	0.64	0.65
P, % of DM	0.36	0.43
NEl，MCal/kg	1.50	1.81

[1] DDGS= Distillers Dried Grains with Solubles; CP = crud protein; NDF = neutral detergent fiber; ADF = Acid detergent fiber; NFC= non fibrous carbohydrates

[2] Concentrates：(% of DM) CP 34.00%，NPN (nonprotein nitrogen) 7.36%，NDF 15.22%，ANF 6.15%，

EE(ether extract)7.2%，NFC 36.13%，Ash 8.42%，

[3] Premix：water 9.34%，Ca 3.22%，P 0.83%，Mg 0.69%，K 1.01%，S 0.31%，Na 1.84%, Cl 2.25%，Fe 112.00 mg/kg DM， Zn 700.00 mg/kg DM，Cu 140.00 mg/kg DM，Mn 653.34mg/kg DM，I 9.33 mg/kg DM，Se 3.08 mg/kg DM，Co 9.35 mg/kg DM，Vitamin A106,720 IU/ kg DM，Vitamin D 13,280 IU/kg DM，Vitamin E 320 mg /kg DM。

Table S2 Primers used for real-time PCR quantification

Target bacteria	Primer	Tm (°C)	Product size(bp)	Reference
General Bacteria 16Sr DNA	F: 5'- CGGCAACGAGCGCAACCC -3'<br>R: 5'-CCATTGTAGCACGTGTGTAGCC -3'	58	130	[1]
Ruminococcus albus	F: 5'-CCCTAAAAGCAGTCTTAGTTCG-3'<br>R: 5'-CCTCCTTGCGGTTAGAACA-3'	54	176	[2]
Ruminococcus flavefaciens	F: 5'-CGAACGGAGATAATTTGAGTTTACTTAGG-3'<br>R: 5'-CGGTCTCTGTATGTTATGAGGTATTACC-3'	58	132	[2]
Fibrobacter succinogenes	F: 5'-GGAGCGTAGGCGGAGATTCA-3'<br>R: 5'-GCCTGCCCCTGAACTATCCA-3'	59	97	[2]
Butyrivibrio fibrisolvens	F: 5'-ACCGCATAAGCGCACGGA-3'<br>R: 5'-CGGGTCCATCTTGTACCGATAAAT-3'	59	124	[2]
Prevotella ruminicola	F: 5'-GCGAAAGTCGGATTAATGCTCTATG-3'<br>R: 5'-CCCATCCTATAGCGGTAAACCTTTG-3'	59	78	[2]
Selenomonas ruminantium	F: 5'-GGCGGGAAGGCAAGTCAGTC-3'<br>R: 5'-CCTCTCCTGCACTCAAGAAAGACAG-3'	60	83	[2]
Megasphaera elsdenii	F: 5'-GACCGAAACTGCGATGCTAGA-3'<br>R: 5'-CGCCTCAGCGTCAGTTGTC-3'	58	129	[2]
Veillonella alkalescens	F: GACGAAAGTCTGACGGAGCA<br>R: TGCCACCTACGTATTACCGC	60	132	This study

Table S3 The percentage of reads mapped to each enzyme involved in propionate propionate

Enzyme	HF		LF		SEM[3]	*P*-value[4]		
	BF0h[1]	AF4h[2]	BF0h	AF4h		Feed	Time	Feed*time
EC:1.1.1.27	0.2186	0.2843	0.1133	0.1169	0.0056	NS	NS	NS
EC:1.3.8.7	0.5443	0.4514	0.7133	0.4702	0.0044	NS	*	NS
EC:2.8.3.1	0.0078	0.0039	0.0958	0.0674	0.0002	**	NS	NS

[1] BF(0), before feeding (0 h); [2] AF(4), after feeding (4 h); [3] SEM for feed × time; [4] NS, not significant ($P > 0.05$); *, ($P < 0.05$); **, ($P < 0.01$).

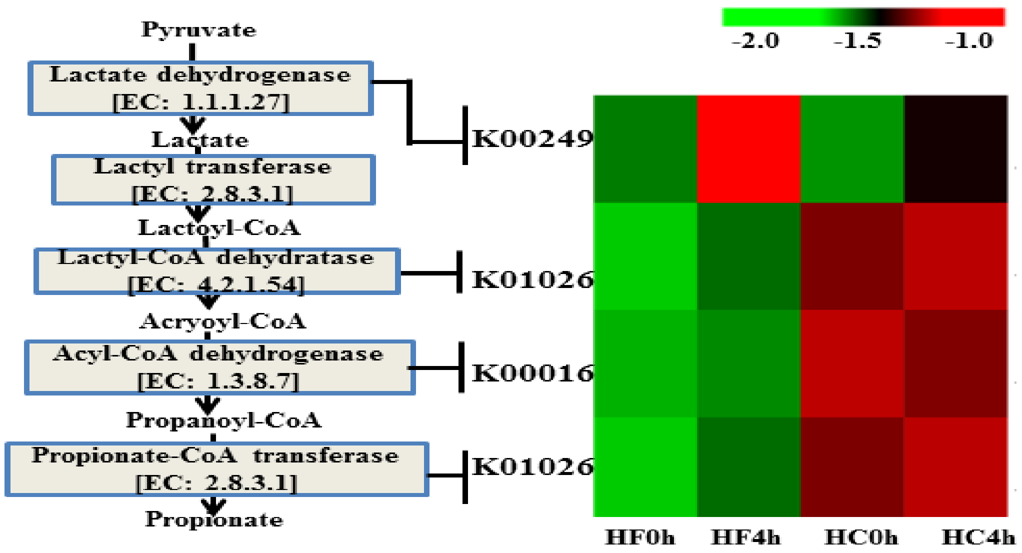

Figure S1 The metagenomic abundance of key elements of the propionate production pathway. Left pane: the propionate production pathway, showing enzyme classification (EC) numbers. Right pane: heatmap of KEGG orthologues for the EC numbers involved in propionate production (lines connect the heatmap to the propionate production pathway indicating which K0 numbers represent the given enzymes); EC:4.2.1.54 No reference genes for this function were available in the KEGG database. Green and red color indicates low and high abundance, respectively. Computation based on Log10.

References:

[1] McSweeney, C.S., Palmer, B., Bunch, R., Krause, D.O. Effect of the tropical forage calliandra on microbial protein synthesis and ecology in the rumen. *J Appl Microbiol*. 2001, 90(1), 78-88.

[2] Khafipour, E., Li, S., Plaizier, J.C., Krause, D.O. Rumen microbiome composition determined using two nutritional models of subacute ruminal acidosis. *Appl Environ Microbiol*. 2009, 75, 7115-24.
